# 5-Aminolevulinic Acid and Red Led in Endodontics: A Narrative Review and Case Report

**DOI:** 10.3390/gels8110697

**Published:** 2022-10-29

**Authors:** Simonetta D’Ercole, Teocrito Carlesi, Tatiane Cristina Dotta, Tania Vanessa Pierfelice, Emira D’Amico, Domenico Tripodi, Giovanna Iezzi, Adriano Piattelli, Morena Petrini

**Affiliations:** 1 Department of Medical, Oral and Biotechnological Sciences, University of Chieti-Pescara, 66100 Chieti, Italy; 2 Department of Dental Materials and Prosthodontics, School of Dentistry of Ribeirão Preto, University of São Paulo, São Paulo 14040-904, Brazil; 3 School of Dentistry, Saint Camillus International University for Health Sciences (Unicamillus), 00131 Rome, Italy; 4 Fondazione Villa Serena per la Ricerca, 65013 Città Sant’Angelo, Italy; 5 Casa di Cura Villa Serena, 65013 Città Sant’Angelo, Italy

**Keywords:** photodynamic therapy, red led, 5-aminolevulinic acid, endodontics

## Abstract

The present study aims to discuss the main factors involving the use of 5-aminolevulinic acid together with red LED light and its application in endodontic treatment through a narrative review and a case report. Persistence of microorganisms remaining on chemical-mechanical preparation or intracanal dressing is reported as the leading cause of failure in endodontics. Photodynamic therapy has become a promising antimicrobial strategy as an aid to endodontic treatment. Being easy and quick to apply, it can be used both in a single session and in several sessions, as well as not allowing forms of microbial resistance. 5-aminolevulinic acid in combination with red LED light has recently been studied in many branches of medicine, with good results against numerous types of bacteria including *Enterococuss faecalis*. The case report showed how bacterial count of CFU decreased by half (210 CFU/mL), after 45 min of irrigation with a gel containing 5% of 5-aminolevulinic acid compared to the sample before irrigation (420 CFU/mL). The subsequent irradiation of red LED light for 7 min, the bacterial count was equal to 0. Thus, it is concluded that the use of 5-aminolevulinic acid together with red LED light is effective in endodontic treatment.

## 1. Introduction

### 1.1. Photodynamic Therapy

Biological systems are influenced by visible light because of some chromophores inside the cells can absorb it, so they can be considered as photosensitizers. A photosensitizer (PS) is a compound capable to induce cytotoxic effects in target cells upon its photoactivation at specific wavelength ranges. Because of these properties, PS are utilized for therapeutic purposes as known photodynamic therapy (PDT). Most of these photosensitizer compounds emit the energy from their first excited singlet state as fluorescence that permits to use them also for diagnostic purposes [1,2].

On the base of the purposes, in the literature PDT can be distinguished into various subgroups: photodynamic antimicrobial therapy (aPDT) [3], photodynamic antimicrobial chemotherapy (PACT) [4], photodynamic disinfection (PDI) [5], antimicrobial photodynamic inactivation (aPDI) [6], lethal photosensitization [7] and photoactivated disinfection (PAD) particularly used in dentistry field [8].

The antimicrobial activity of PDT is due to the presence of positively charged functional groups that characterize specific PS-like phenothiazines such as toluidine blue and methylene blue [9], porphyrins, phthalocyanines and natural products such as chlorophyllin [10], curcumin and hypericin [11], each photoactivated at specific absorption wavelengths.

The efficacy of PDT as antimicrobial strategy was first introduced in 1900 by Oscar Raab, who reported the lethal effect of acridine hydrochloride on *Paramecium caudatum* [12], whereas the name “photodynamic” was introduced later by von Tappeiner [13].

Recently, numerous research studies have been resumed to evaluate the efficacy of photodynamic therapy in various medical specialties [14,15,16,17] including severe tumor pathologies and premalignant diseases [18], as it appears to be a selective therapy and non-invasive or moderately invasive treatment.

**Figure 1:**
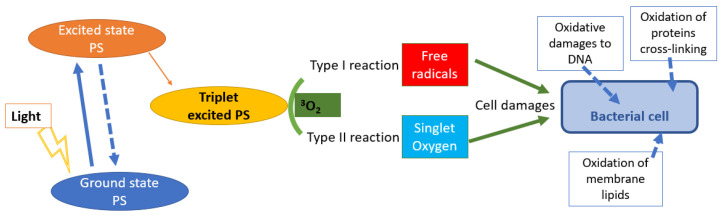
The multiple mechanisms that are triggered by PDT and that kill micro-organisms.

PDT has demonstrated its efficacy against localized infections by bacteria, viruses and fungi [3], and represents an excellent alternative strategy to fight antibiotic resistance, since the development of a resistance to photodynamic protocol is difficult because in microbial cells the action of singlet oxygen and free radicals, produced during PDT, act on different cellular structures (Figure 1) [3], as it is known singlet oxygen reacts with proteins, nucleotides and lipids. Considering the higher solubility of singlet oxygen in lipids than in aqueous environments, and the higher proportion by mass of lipids in the cellular membrane contribute to make biological membranes the most attractive targets. Thus, the consequent oxidation and peroxidation of lipids become determinant in the inactivation of microorganisms. In addition, the diversity of ROS and their high reactivity towards different biomolecules ensures that PDT is a multi-target approach to control infectious diseases, which reduces the efficacy of drug resistance mechanisms. In addition, this multiple attack, unlike antibiotics, represents an advantageous action against oral biofilms [3,19].

PDT consists of two basic steps: the accumulation of a photosensitizing agent inside target cell and its subsequent activation by exposure to a visible light with a specific wavelength leads to a change in the state of energy of the photosensitizing agent from a low singlet “ground state” to a high energy “triplet state” [20,21].

In the presence of oxygen, two different mechanisms of action of the chemical interaction with biomolecules can occur: production of free radicals (type 1) and of singlet oxygen (type 2). Free radicals (ROS) such as peroxide anions, superoxide and hydroxyl radical can be directly responsible for cell damage [22,23].

Singlet oxygen is a hyperactive oxygen able to react with the substrates within its microenvironment with a range of action limiting to 30 nm from the area where the photosensitizer is present [19,24]. Often both type 1 and type 2 mechanisms can occur, thus it is not easy to distinguish them, it depends a lot on the amount of the photosensitizing agent and the oxygen [19,25]. Therefore, the interaction between light, photosensitizer and oxygen is the basis of the photodynamic action [26].

Cell death by photodynamic therapy is due to damages to the DNA, or to the cell plasma membrane caused by an altered functioning of the exchange and transport systems, by cancellation of the function of enzymes such as lipid peroxidation, as illustrated in Figure 1 [4,27,28,29,30].

### 1.2. Photodynamic vs. Photoinactivation Therapy

Several authors have shown how the use of red or infrared light alone can have some antibacterial properties, but non-lethal effects, on some Gram-negative and Gram-positive bacteria, such as *Pseudomonas aeruginosa* and *Enterococcus faecalis* [31,32,33,34].

The photoinactivation mechanism is induced after the photoinactivation of an endogenous substance inside bacterial cells by producing free radicals which determine cytotoxic action [35]. It is important to underline that the quantity of photosensitive endogenous substances differ according to the type of bacterial strain in variable manner.

Instead, as previously described, photodynamic therapy requires the use of an exogenous photosensitizing agent that is up-taken by target cells to induce enough photosensitizer to induce the cytotoxic effect [36,37]. Photodynamic therapy and photoinactivation should not be confused with the use of high-power lasers which lead to the complete elimination of the percentage of bacteria due to the high temperature generated [38,39]. There are numerous studies in the literature, including in the field endodontics, which use low-power lasers to avoid necrosis and damage to healthy dental tissues [40,41], in which cause minimal temperature variation, nonetheless, their effectiveness is possible when used with a photosensitizing agent [40,42].

The types of lasers and their powers used in endodontics are described in Table 1. Among in vitro and in vivo studies, there is a great variety in the protocol for using the laser. The diode type is one of the most used, with a wavelength ranging from 635 to 940 nm [43,44,45,46,47,48,49]. The power of the LED light varies between 265 to 880 nm [31,33,50,51,52], and the Helbo laser with a wavelength of 660 nm [44]. In addition, the duration of application ranged from 10 s to 20 min.

### 1.3. Specific Wavelength for Specific Photosensitizers

There are many natural and synthetic photoactive agents that can have a photoactivating action; the most studied with antimicrobial activity are halogenated xanthines, acridine, conjugated chlorides and phenothiazines [3]. An ideal photosensitizing agent that induces good photodynamic action should have some important characteristics in addition to the most common photophysical properties, such as a good quantum yield of triplet formation, singlet oxygen production, but also a high degree of purity, a high molar extinction coefficient and absorption in the red region of the spectrum and a high quantum yield of triplet state formation with relatively long half-life (µ,s) to ensure efficient formation of ROS (radical oxygen species, free radicals). Furthermore, the ideal photosensitizer should be non-toxic in the absence of light and have an excellent accumulation selectivity in a target cell or tissue to direct the photodynamic process to a specific site [56].

A good photosensitizer should have a broad spectrum of action (bacteria, fungi and protozoa), selectivity of damage with respect to the host tissue or cell, lack of development of resistance to photodynamic therapy, lack of mutagenicity and activation by low-cost light sources [57].

The selective accumulation capacity of the photosensitizing agent depends on its chemical-physical characteristics but also on the physiology and morphology of the target cell or tissue. Bacteria can be classified according to the characteristics of the external coating which makes them more or less sensitive to photoactivation. Gram-positive bacteria are more vulnerable to photodynamic therapy because they have a more porous cytoplasmic membrane formed by peptidoglycan and lipoprotein acid which allows the passage of the photo activator [3,58]. Gram-negative bacteria have a double membrane, one internal and one external, which is much more difficult to cross. An important biological property of the outer membrane is toxicity. This property depends on the portion represented by the “lipid A” of the lipopolysaccharide. Unlike the cytoplasmic membrane, the outer one, despite being a lipid bilayer, is partially permeable to small molecules [59]. This occurs thanks to the presence of small proteins called porins, which act as channels; solutes with suitable characteristics such as hydrophilicity and molecular weight lower than 600–700 [60] that can perfuse it. To be effective, the photosensitizing agent must pass through the cell membrane or be transported into the cytoplasm, inhibiting the synthesis of proteins, DNA and RNA [58]. To achieve a good antibacterial efficacy, each photosensitizing agent must be excited at a precise and correct wavelength of visible light measured in nanometers [61].

In endodontics, different photosensitizing agents have been studied in combination with different sources of activation lights and the results have been controversial [42,53,62].

Given the limited number of studies that have used the same photosensitizing agent and the same activation light with different types of study protocols, it is difficult to compare them [63]. The most used photosensitizing agents are phenothiazines, hematoporphyrin derivatives, cyanine, phthalocyanines, phytotherapy and chlorine, which have a wavelength of maximum absorption ranging from 550 nm of phytotherapies up to a maximum of 805 nm of cyanine [61,63,64,65].

The agents derived from phenothiazine compounds, such as methylene blue and toluidine blue, are certainly the most used and studied; the first one has a wavelength of maximum absorption at 660 nm, while the second one at 630 nm [66,67,68]. Both have expressed bactericidal abilities in both Gram-positive and negative bacterial strains [69], such as *Enterococcus faecalis* [8,70,71,72,73,74]. Due to their hydrophobic and hydrophilic characteristics, respectively, some authors indicate these phenothiazine-based dyes as suitable for photodynamic therapy in endodontics [58,74,75,76].

Three different types of light sources are documented in the literature to perform photodynamic therapy in endodontics: laser, halogen light and light-emitting diodes (LED) [69,77]. Different types of lasers such as helium-neon, argon, gallium-arsenide diode, respectively, red, blue or infrared, have been also used and their wavelength of maximum absorption ranging from 630 to 800 nm [78].

In detail, diode lasers are mainly used for their low cost, because they emit less heat and specially, they are equipped with a fiber that can be inserted inside the root canal [63].

Halogen lamps are most common in dental practice, they have the advantage of being able to apply the filters necessary for the photoactivating agent but the disadvantage of generating heat. The halogen lamps are not equipped of a fiber able to convey the light inside the channel, but they have only tips [69].

LED lamps also have lower costs than lasers, they are widely used in photodynamic therapy especially for easily accessible tissues, such as in dermatology [29,79,80].

**Figure 2:**
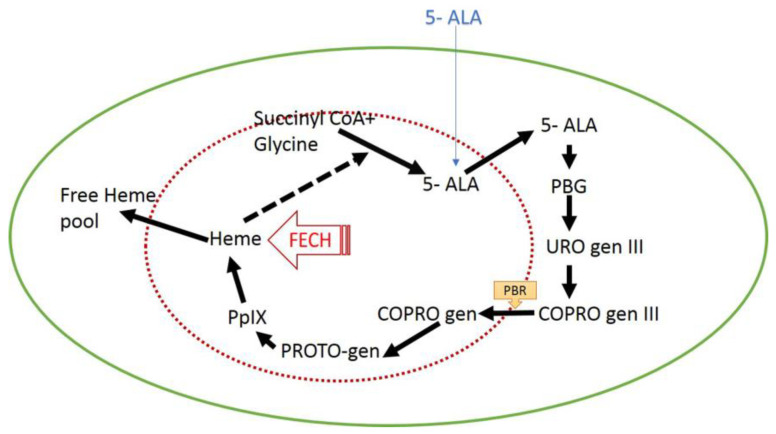
Illustration of the heme pathway that occurs in part inside mitochondria and in part in the cytosol. 5-aminolevulinic acid (5-ALA) is converted through different steps into Protoporphyrin IX (PpIX) that in turn is the precursor of heme. The conversion from PpIX into heme takes place by ferrochelatase enzyme (FECH).

Photodynamic therapy was less effective against Gram-negative bacteria due to the structure of the external membrane which is less permeable to photoactivating agents; on the contrary, more effective actions have been found against Gram-positive bacteria, due to their more porous membrane structure [19,58,81]. The mechanism of action of photodynamic therapy based on the production of singlet oxygen and related ROS production, inducing irreversible damage to the bacteria [82,83]. In detail, a photosensitizer such as Protoporphyrin IX (PpIX) was accumulated in bacteria because of the lack of specific enzyme such ferrochelatase (Figure 2). Therefore, bacteria accumulated a greater amount of PpIX that stimulated the production of singlet oxygen and ROS with consequently cytotoxic effect [84].

### 1.4. Photodynamic Therapy Based on 5-Aminolevulinic Acid and Red LED

5-aminolevulinic acid (5-ALA) is an intrinsic photosensitizing agent different from phenothiazine derivatives such as toluidine blue and methylene blue, as it is converted in situ into an endogenous substance essential for the formation of the heme group, protoporphyrin IX (PpIX) a powerful photosensitizing agent [85]. This ALA photosensitizing agent and its derivatives have been used for many years with excellent efficacy in antibacterial therapy and in many cancerous and pre-cancerous diseases, in dermatology and in disease of the oral cavity [86,87,88,89]. Moreover, it is a natural molecule that has been extensively studied in other research fields such as agriculture and biotechnology [90].

5-ALA is up-taken by all types of cells, but in healthy cells the substance is metabolized through many steps into the HEME, while in cancerous and bacterial cells it remains accumulated as the precursor of HEME such PpIX [85,91]. The greater bactericidal efficacy of 5-ALA derives, on the other hand, following the photoactivation of accumulated PpIX and the consequent ROS production that induce cell death [92].

PpIX must be activated by light, and in particular red light with a wavelength of about 630 ± 10 nm was found to be effective in the photoactivation of PpIX by causing cytotoxic reactions in the presence of oxygen in bacterial and cancerous cells [91,93].

**Figure 3:**
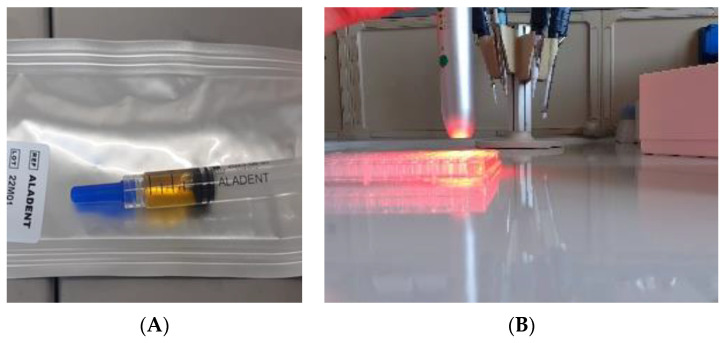
(**A**) ALADENT gel into the syringe is a yellow liquid that becomes gel at the temperatures above 28 °C. This property favors its topical application. (**B**) The light source used is AlGaAs power 630 nm ± 10 nm LED device FHWM nm emits a red light visible to eyes.

“Aladent” (ALAD) (Alphastrumenti Srl, Melzo (MI), Italy), a registered trademark covered by a patent, it is a gel that contains 5% of 5-aminolevulinic acid and some authors have shown that it has antibacterial properties [92,94]. This product is liquid, but it gels at the temperature above 28 °C, its formulation includes a poloxamers mixture that confer good muco-adhesive properties that make this thermos-gel an ideal product for oral application (Figure 3). Furthermore, ALAD contains some preservatives such as sodium benzoate and potassium sorbate, which would be able to induce some quantities of free radicals with a bactericidal potential [95,96]. In particular, the antimicrobial action of ALAD has been shown to be effective against various types of both Gram-negative and Gram-positive bacteria such as: *Staphylococcus aureus, Enterococcus faecalis, Escherichia coli, Veillonella parvula*, *Porphyromonas gingivalis* and *Pseudomonas aeruginosa* [31,32,88,97]. It has also been shown to be effective against *Candida albicans* [98]. The presence of specific preservatives and an acid pH of about 3.50 of the ALAD gel seems to give an antibacterial efficacy towards the Gram-negative ones more than other compounds based on 5-aminolevulinic acid [88,98,99]. The ALAD-PDT protocol includes the administration of ALAD gel for 45 min and the consequent irradiation with 630 nm red light for 7 min. The light source used a power LED device TL-01(ALPHA Strumenti, Melzo, Italy) that has a single emitted LED with 6 mm diameter, and it emits a red light visible to eyes as illustrated in Figure 3B. The exit and surface irradiance are 380 mW/cm^2^ and the total specific dose is 23 J/cm^2^ for each minute of irradiation, as previously described by Radunovic M et al. [88].

The combination of the photosensitizer and the light is necessary to obtain the complete inactivation of bacteria, as was shown by Radunovic in 2020 and Petrini in 2022 [88,97]. A solution containing 50% of ALAD applied for 45 min in combination of the light at 630 nm for 7 min produced a total inactivation and an evident lethal effect on *E. faecalis* (95% dead cells) [88].

Based on in vitro studies conducted on the efficacy of the bactericidal action of ALAD gel and LED red light against *Enterococcus faecalis* and based on the results obtained with a LED lamp (Fotosan) equipped with fiber and toluidine blue in photodynamic therapy in endodontics, some authors have proposed a fiber system connected to the LED light to optimize the application of light inside the root canals [53,100].

## 2. Case Report

To better understand the action of 5-aminolevulinic acid and red led in endodontic treatment, a demonstrative clinical case is reported.

**Figure 4:**
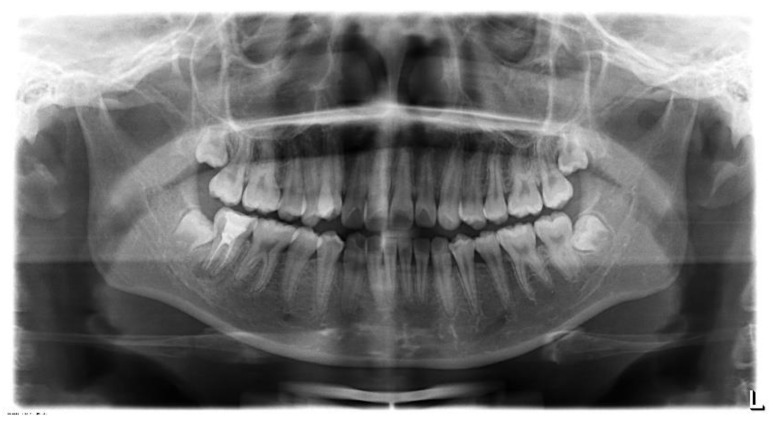
X-ray orthopantomography of the patient.

**Figure 5:**
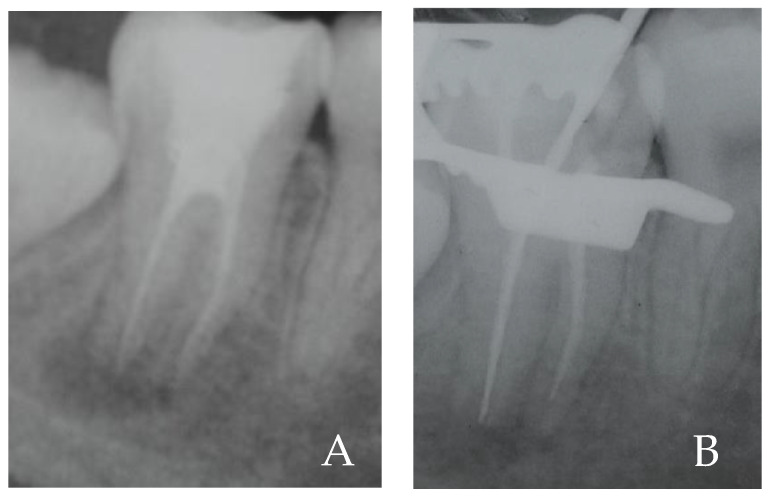
(**A**) Dental element 47 with radiolucent image in the periapical region. (**B**) Retreatment of inferior second right molar, with periapical lesion, after working length confirmed by intraoperative X-ray.

A young Asian woman, 20 years old, was referred for an examination on tooth 47 (Figure 4). The patient reported having previously had swelling. A periapical radiograph on the tooth, in which it was endodontically treated, showed a radiolucent image in the periapical area (Figure 5A). A diagnosis of chronic apical periodontitis with a recent episode of abscess was performed involving tooth 47. Therefore, it was decided to perform a non-surgical retreatment that the patient agreed by signing the consent to the treatment. Thus, after the application of anesthesia, the rubber dam was placed, which was disinfected.

Under an intra-operative microscope, a disassembly of the old composite resin restoration was carried out using a turbine-mounted diamond bur with water irrigation. The chamber opening was obtained, and the old root canal filling (carrier base filling) was removed with NiTi Mtwo instruments (Sweden Martina, Padova, Italy). The working length of 18 mm was measured with an electronic device (Apit, Osada, Japan) and confirmed by an intra-operative periapical X-ray, with a 25.06 NiTi Mtwo instrument (Figure 5B). The discharge of purulent material and pus from the canal after reaching the working length is noted.

**Figure 6:**
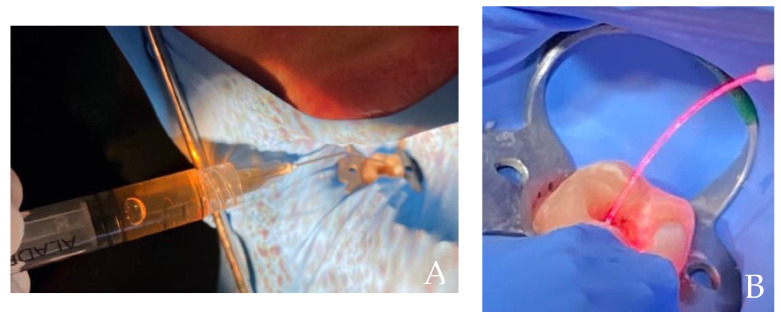
(**A**) ALAD gel inserted into the canal. (**B**) After 45 min, photoactivation by red light, of the gel containing 5% of 5-aminolevulinic acid was performed by a special intracanal fiber for 7 min.

In this initial phase, no irrigate solution was used, and washes were performed with a sterile physiological solution. Samples were collected to carry out the CFU bacterial count using 3 sterile paper cones 30.02 ISO (Table 2—sample 1). The Aladent gel (ALAD), containing 5% of 5-aminolevulinic acid was then inserted by means of a thin syringe inside the canals (Figure 6A), and brought to working length by means of sterile paper cone 30.02. After 45 min, in which the rubber dam was not removed, the second sampling was performed (Table 2—sample 2). Immediately after, photoactivation was performed inside the canal by means of a red LED lamp (630 nm) through a dedicated optic fiber which was brought to working length and an up and down movement was performed within the canal for 7 min (Figure 6B). After the photo-activation, the third sampling was performed inside the root canal (Table 2—sample 3). The paper cones of the samples were stored in sterile pipettes with physiological solution, closed with an airtight cap and immediately taken to the microbiology laboratory to perform the bacterial count CFU/mL. As can be seen from the data shown in Table 1, the bacterial count already, only at the second sample 2, without activation, halved the bacterial count inside the canal; after the activation of 7 min the bacterial presence was equal to 0 (sample 3).

Only after having performed the third sampling, it was washed with 2.5 mL of sodium hypochlorite NaOCl 5% and ethylenediamine tetra-acetic acid solution, EDTA 17% (Ogna Laboratori Farmaceutici, Muggiò, MB, Italy). After drying the canals with paper cones, it was dressed with calcium hydroxide and a temporary restoration was performed.

**Figure 7:**
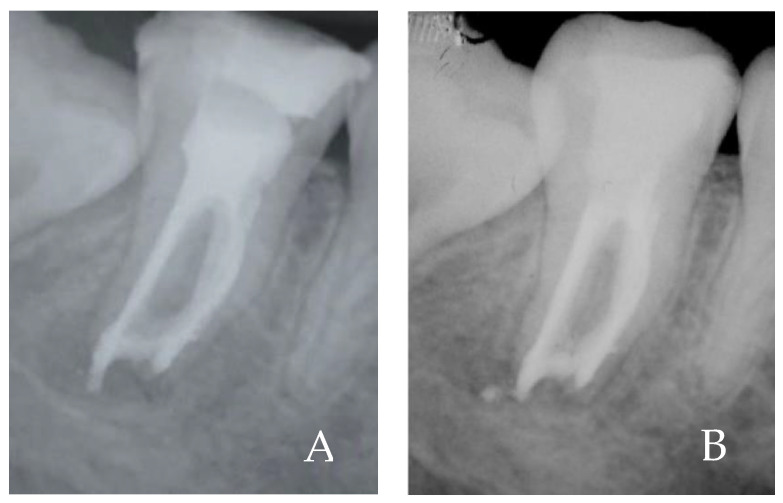
(**A**) Post-operative X-ray with root canal obturation with pre-plasticized gutta-percha. (**B**) 4-month follow-up X-ray showing good healing.

After 2 weeks, the patient was seen again, and she reported no pain or edema. The classic root canal treatment was continued, performing apical finishing up to diameters of the Mtwo 50.04; canal washes were performed with NaOCl and EDTA and after drying the canals, root canal obturation was performed using preplasticized gutta-percha, Microsel (Sweden Martina, Padova, Italy) according to McSpadden technique. Post obturation root canal, intraoperative X-ray was performed (Figure 7A). Direct reconstruction with composite resin was also performed in the same appointment. The control radiograph at 4 months of follow-up shows radiographic signs of healing of the periapical radiolucency (Figure 7B), and the patient reports has not experienced any discomfort.

## 3. Photodynamic Therapy in Endodontics

The persistence of bacterial microorganisms and their toxins are the cause of dental pulp pathologies. Their elimination and decontamination are a fundamental step for the long-term success of endodontic treatment [101]. Different bacterial types are able to organize a bacterial biofilm that can nest in areas of the root canal system that are difficult to access for disinfection, this can cause the root canal treatment to fail [102].

The total elimination of the bacterial load in the root canal system is the real challenge of the dentist. The chemo-mechanical instrumentation, the basis of root canal treatment, consists of the use of mechanical or manual instruments in addition to irrigants, such as sodium hypochlorite, EDTA acid, used alone or combined with other types of irrigants such as chlorhexidine and medicinal pastes such as calcium hydroxide [68]. Although these techniques are reported to be the gold standard, complete elimination of the bacterial load within root canals is not reached [103] due to the complexity of the anatomy of the root canal system [44].

In primary endodontic infections the biofilm was produced by Gram^−^ anaerobic bacteria, while in secondary infections biofilm was also associated with Gram^+^ bacteria [104]. The bacteria most found in the roots already treated with persistence of infection is surely *Enterococcus faecalis*, a Gram^+^ facultative bacteria [105]. This bacterial microorganism, due to some of its specific skills, can survive even in unfavorable microenvironments with few nutrients, and large variations in pH, temperature and quantity of oxygen, and these characteristics make its eradication difficult [106,107,108]. Therefore, due to the permanence of bacteria inside the canals, various techniques have been investigated to implement the antibacterial action inside the root canals, such as photodynamic therapy [109].

In recent years there has been a tendency to perform more conservative mechanical preparations thanks to the aid of various systems and drugs that improve and tend to replace the traditional chemo-mechanical disinfection in endodontics. Several authors have proposed photodynamic therapy in the treatment of teeth with periapical lesions in primary treatments [110,111,112], but above all in teeth already endodontically treated (retreatments) [47,53,62,113,114]. An almost total reduction of up to 99% of CFUs was achieved in studies based on the use of infected extracted teeth [115,116].

Several studies have been conducted that have verified the efficacy of photodynamic therapy in endodontics, in vivo, in vitro and ex vivo studies. However, in the literature, the efficacy of photodynamic therapy in endodontics is still a controversial and highly debated topic. There are several narrative reviews on this topic in the literature [17,101,117], nonetheless, there are few systematic reviews. In a recent systematic review and meta-analysis of the reading on the efficacy of photodynamic therapy in secondary endodontic infections, only 8 studies, out of 1513, were used in the quantitative analysis of the review, where it was concluded that photodynamic therapy has an efficacy in reducing the bacterial load in endodontic retreatments [118].

## 4. In Vitro and Ex Vivo Studies

As previously described, *Enterococcus faecalis*, a Gram-positive anaerobic *cocco* bacteria, is certainly the most commonly encountered species and plays a fundamental role in the persistence of endodontic infections [104,105,106].

The use of photodynamic therapy as an effective action against this important facultative *cocco* has been confirmed by some authors in a systematic review of the literature of in vitro studies [119]. Several authors have reached similar conclusions on the good efficacy of the photodynamics therapy in root canals infected with *Enterococcus faecalis* [8,47,62,70,71,72,73,74,109,110,111,120,121,122,123,124,125].

5-aminolevulinic acid [88,98], methylene blue [47,72], methylene blue with added nanoparticles [120], toluidine blue [47], erythrosine [126] and indocyanine green [125,127,128,129] are the photosensitive agents with different applications of light sources which have shown positive effects on the antibacterial action against *Enteroccocus faecalis*.

Some authors have shown that if photodynamic therapy is performed in addition to the use of 5% sodium hypochlorite NaOCl, there is an almost absolute reduction of 99.99% in the bacterial load of *Pseudomonas aeruginosa*, *E. faecalis* and *Staphylococcus aureus* [130]. Bacterial counts resulted diminished in numerous studies based on the use of photodynamic therapy; some authors have also compared these results with the use of sodium hypochlorite and standard chemo-mechanical therapy [122,131,132,133,134].

Low percentages of 0.5 and 2.5% sodium hypochlorite NaOCl did not give better results than photodynamic therapy alone [73,135], while the common action of adding chemo-mechanical and photodynamic therapy was found to have better efficacy against antibacterial action in root canals [111,122,133,136].

Some authors, in ex vivo studies, have shown that chemo-mechanical root canal therapy, with irrigation of standard 5% sodium hypochlorite, achieved greater efficacy than photodynamic therapy [135,136,137]. Photodynamic therapy did not show ameliorative effects on bacterial counts with respect to chemo-mechanical therapy with 2.5% sodium hypochlorite [136,138].

It is important to underline that the success of the photodynamic protocols is linked the presence of sufficient oxygen. Thus, a low amount of oxygen present in the root canal system and in the dentinal tubules could represent a limit of the photodynamic performance in endodontics. To overcome this limitative aspect is fundamental to add an adequate diffusion of the photosensitizing agent molecules within this micro-anatomy

Bioactive micro and nanoparticles in photosensitizing agents is also being evaluated to improve the effectiveness of photodynamic therapy including bacterial biofilm [120,127].

## 5. In Vivo Studies

There are several studies in the literature performed in vivo on patients; many of these have shown how photodynamic therapy in addition to conventional chemo-mechanical therapy reduces the bacterial load inside the root canal [11,44,45,47,51,53,110,123,124,138,139]. In contrast, two studies on adults and one on deciduous teeth, have demonstrated a similar reduction in bacterial load between the various comparison groups, conventional therapy with and without the addition of photodynamic therapy [46,53,54,140]. Garced et al. in his study, conducted on patients presenting periapical lesions, demonstrated how polyethyleneimine conjugate and chloro-e6 activated by laser fiber, increased the effectiveness of the traditional chemo mechanical therapy. Furthermore, if the treatments were performed in two sessions in which an intermediate dressing with calcium hydroxide was used, the reduction in the bacterial load with a second intervention of chemo-mechanical treatment and photoactivation, reached 99.9% [111]. Other authors published an important in vivo study in 2018, in which they demonstrated that photodynamic therapy with toluidine blue was effective in decreasing bacterial load in endodontic infections on necrotic teeth not endodontically treated [47].

In 2021 Bharti et al. [51] demonstrated an efficacy of the action of the photodynamic therapy with toluidine blue activated by LED light (fotosan) in teeth with periapical lesions. The control groups, treated with standard treatment and only calcium hydroxide dressing without photodynamic therapy, were found to have greater bacterial colonization of *Enteroccus faecalis*.

Similar bacterial load reduction results were also found in an in vivo study by Garcia-Zorita et al. [112] in which, from a baseline of 113.5 ± 130 CFU/tooth, at the second sample after CMP (chemomechanical preparation) the samples had 26.5 ± 72 CFU/tooth, with a reduction after PDT to 4.2 ± 13CFU/tooth. The microbiota was also identified in this study, and colonies of *E. faecalis* were observed in 16.6% of the first samples (baseline) with a mean of 93 CFU/tooth. After conventional CMP the reduction in CFU was up to 26, whereas PDT achieved a significant additional reduction of 84 CFU, which represents a 90.3% decrease in bacterial count.

In studies in which a similar action was shown between chemo-mechanical therapy with and without photoactivation, and dressing with calcium hydroxide, it is important to underline that they were conducted using methylene blue as a photosensitizer. The use of methylene blue was not accompanied by a light source with a specific wavelength of 660 nm, as recommended by several authors [66,67,68], but by a diode laser fiber with a wavelength of 810 nm. This aspect could be the cause of a similar result between the various groups. In order for the calcium hydroxide dressing to be effective it is necessary to perform the treatments in several sessions because it must be repeated [141]. Although the results of photodynamic therapy were similar to the control groups, the use of photodynamic therapy could be useful when you want to conclude the therapy in a single session, with greater comfort for the patient.

In a study conducted in pediatric patients aged 2 to 5 years, involving deciduous teeth, it reported that conventional chemo-mechanical treatment combined with antimicrobial photoactivation was effective, but did not show statistically significant efficacy alone conventional endodontic treatment [140]. Methylene blue was also used in this study; nonetheless, the wavelength of the laser light source was according to guidelines of 660 nm, and the authors found a 93% decrease in bacterial load for conventional treatment, compared to 99% of conventional treatment with added photodynamics.

In in vivo studies on patients, some authors specifically assessed the count of *Enterococcus faecalis*, a bacterium most present in resistant endodontic infections. The use of photodynamic therapy in combination with traditional chemo-mechanical treatment had led to a 95% reduction in CFUs [43,44,55].

A histopathological study by Silvia et al. [62] performed in dogs with an induced apical periodontitis showed that PDT-treated groups appeared moderate/severe enlargement of the periapical region without inflammatory cells, moderate neoangiogenesis and fibrogenesis, and smaller periapical lesions. Although apical closure by deposition of mineralized tissue was not achieved, these results suggest that PDT may be a promising adjunct therapy to cleaning and shaping procedures in teeth affected by apical periodontitis treated in a single session.

Photodynamic therapy with the addition of a chelating agent such as citric acid, has a cleaning and disintegrating action against the biofilm, similar to 2.5% sodium hypochlorite. Similarly, the conventional treatment with citric acid and 2.5% sodium hypochlorite and photodynamic therapy produced similar results [139].

In vitro, it has been shown that 17% EDTA acid for 2 min allows a better penetration of the photosensitizer into the dentinal tubules [142]. This aspect is fundamental because the action of photodynamic therapy is linked to the perfusion of the photosensitizing agent throughout the canal system, otherwise there can be no bactericidal action by this. In addition, the diffusion of light within the canal system is also necessary; for this reason light sources such as laser diode fibers were used, or fibers were introduced in addition to LED lamps [29,79,80].

In their study, Lauritano et al. [143] compared the supportive therapy scaling and root planning (SRP) with and without the addition of ALAD gel in the treatment of chronic periodontitis on 20 patients. They observed that the sites treated with the combination of SRP and ALAD gel showed a significant reduction in the total bacterial loading compared to the treated sites with SRP alone. Furthermore, Rossi et al. [89] also considered the photodynamic therapy based on ALAD gel a support for the conventional treatment, favoring the maintenance of severely compromised teeth and the improvement of compromised implant conditions.

A criticism that emerged from one of the few systematic review and meta-analysis on this topic in the literature [118] is that in in vivo studies, the sampling for microbiological analysis was performed with sterile paper cones. This type of sampling can often remain very approximate due to the endodontic anatomical system, including the inability to enter the dentinal tubules. Some authors [51] have proposed a new method of recovery of biological samples to perform the CFU count, directly using the NiTi root canal instruments which, working on the canal walls at the length of the canal, are processed together with the removal of dentin debris that have excised. Photodynamic therapy in endodontics is a topic widely studied in the literature, but it is very varied. The effectiveness of photodynamic therapy in endodontics is still a debated topic, but it has emerged that certainly in addition to the conventional chemo-mechanical treatment accompanied with sodium hypochlorite and EDTA irrigation, the effectiveness of bacterial decontamination of root canals has increased [144,145].

The action of a correct photodynamic therapy is based on a maximum diffusion of the photosensitizing agent within the micro-anatomies of the endodontic system, but a certain amount of oxygen is required, as well as a light source with a certain wavelength and intensity, that must arrive in the areas where the photosensitizer is present.

Synthetic photosensitizing agents, such as phenothiazines, are the most used, but can have discoloration problems [146] if the right doses are not respected or if the pre-irradiation times are too long. 5-aminolevulinic acid and red LED light has recently been studied in many branches of medicine, with good results against numerous types of bacteria including *Enterococcus faecalis*.

Lately, the introduction in bioactive micro and nanoparticles in photosensitizing agents is also being evaluated to improve the effectiveness of photodynamic therapy including against the bacterial biofilm [57,120,127]. All the in vivo studies with promising results were summarized in Table 3.

To optimize and clarify the protocols of a correct photodynamic therapy [115,146], further well-constructed randomized studies are still needed [29,118] to improve the protocols for the use of photodynamic therapy, that resulted a valid aid to traditional chemo-mechanical therapy in endodontics.

## 6. Conclusions

Supported by this narrative review and case report, it is concluded that using a gel containing 5% of 5-aminolevulinic acid and red LED light is a promising antimicrobial therapy to aid endodontic treatment.

## Figures and Tables

**Table 1:** The light-source characteristics and irradiation parameters used by the studies of PDT in Endodontics.

Light Source	Wavelength (nm)	Irradiation Time (s/m)	Authors/Year
Diode laser	660	240 s	Garcez et al., 2010 [43]
Helbo laser	660	60 s	Juric et al., 2014 [44]
Diode laser	660	360 s	Garcez et al., 2015 [45]
Diode laser	810	40 s	Asnaashari et al., 2016 [46]
Diode laser	665	240 s	Asnaashari et al., 2016 [53]
LED	880	5, 10 and 20 min	D’Ercole et al., 2016 [33]
Diode laser	810	10 s	Ahangari et al., 2017 [54]
LED	630	60 s	Asnaashari et al., 2017 [50]
Diode laser	635	30 s	Pourhajibagher et al., 2017 [55]
LED	880	5 min	Petrini et al., 2019 [31]
LED	628	60 s	Bharti et al., 2021 [51]
Diode laser	662	40 s	Manukyan & Risovanniy, 2021 [48]
Diode laser	940	60 s	Dragana et al., 2022 [49]
LED	265 and 280	30, 60 and 90 s	Morio et al., 2022 [52]

**Table 2:** CFU after working length = 420 CFU/mL (sample 1); CFU after 45 min of incubation with Aladent (ALAD) = 210 CFU/mL (sample 2); CFU after 45 min of incubation with ALAD gel and subsequent 7 min irradiation by LED (ALAD-PDT) = 0 CFU/mL (sample 3).

In Vivo Test	CFU/mL
1 sample (After working length)	420
2 sample (After ALAD 45 min)	210
3 sample (After ALAD/LED 7 min)	0

**Table 3:** In vivo studies with promising results.

Garced et al., 2008 [111]	Polyethyleneimine Conjugate and Chloro-e6 Activated by Laser Fiber, Increased the Effectiveness of the Traditional Chemo Mechanical Therapy
Pourhajibagher and Bahador, 2018 [47]	PDT with toluidine blue was effective in decreasing bacterial load in endodontic infections on necrotic teeth not endodontically treated
Bharti et al., 2021 [51]	PDT with toluidine blue activated by LED light was efficacy in teeth with periapical lesions
Garcia-Zorita et al., 2019 [112]	PDT induced a reduction in bacterial load
Okamoto et al., 2020 [140]	CMP combined with antimicrobial photoactivation was effective, but not statistically significant efficacy alone conventional endodontic treatment
Silva et al., 2012 [62]	PDT may be a promising adjunct therapy to cleaning and shaping procedures in teeth affected by apical periodontitis treated in a single session
Lauritano et al., 2022 [143]	Sites treated with the combination of SRP and ALAD gel showed a significantly reduced total bacterial loading compared to the SRP treated sites
Rossi R et al., 2022 [89]	ALAD-PDT was a support for the conventional treatment

## Data Availability

Not applicable.
